# Anti-fungal activity, mechanism studies on α-Phellandrene and Nonanal against *Penicillium cyclopium*

**DOI:** 10.1186/s40529-017-0168-8

**Published:** 2017-03-14

**Authors:** Ji-hong Zhang, He-long Sun, Shao-yang Chen, Li Zeng, Tao-tao Wang

**Affiliations:** 1 School of Chemical Engineering, Xiangtan University, Xiangtan, 411105 People’s Republic of China; 2 College of Horticulture Forestry&Sciences, Huazhong Agricultural University, Wuhan, 430070 People’s Republic of China

**Keywords:** *P. cyclopium*, Antifungal activity, SEM, Mechanism, Membrane permeability

## Abstract

**Background:**

Essential oils from plants have been reported to have wide spread antimicrobial activity against various bacterial and fungal pathogens, and these include α-Phellandrene, Nonanal and other volatile substances. However, biological activities of α-Phellandrene and Nonanal have been reported only in a few publications. Further investigations are necessary to determine the antimicrobial activity of these compounds, especially for individual application, to establish the possible mechanism of action of the most active compound.

**Results:**

The results are shown that α-Phellandrene and Nonanal have a dose-dependent inhibition on the mycelial growth of *Penicillium cyclopium*. The minimum inhibitory concentration (MIC) and minimum fungicidal concentration (MFC) are 1.7 and 1.8 mL/L for α-Phellandrene, 0.3 and 0.4 mL/L for Nonanal, respectively. The volatile compounds altered the morphology of *P. cyclopium* hyphae by causing loss of cytoplasmic material and distortion of the mycelia. The membrane permeability of *P. cyclopium* increased with increasing concentrations of the two volatile compounds, as evidenced by cell constituent release, extracellular conductivity and induced efflux of K^+^. Moreover, the two volatile compounds induced a decrease in pH and in the total lipid content of *P. cyclopium*, which suggested that cell membrane integrity had been compromised.

**Conclusions:**

The results demonstrated that α-Phellandrene and Nonanal could significantly inhibit the mycelia growth of *P. cyclopium* by severely disrupting the integrity of the fungal cell membrane, leading to the leakage of cell constituents and potassium ions, and triggering an increase of the total lipid content, extracellular pH and membrane permeability. Our present study suggests that α-Phellandrene and Nonanal might be a biological fungicide for the control of *P. cyclopium* in postharvest tomato fruits.

## Background

Many plant species, including tomato, synthesize and store numerous volatile terpenoid compounds during normal leaf development (Buttery et al. 1988; Paré and Tumlinson 1999). Tomato is a constitutive emitter of low amounts of mono- and sesquiterpenes under non-stressed conditions, but these emissions become greatly enhanced under stress (Jansen et al. 2009; Maes and Debergh 2003). The volatile blends from *Solanum lycopersicum* leaves detected with SPME GC–MS were mainly terpenoids (i.e., α-Phellandrene), fatty acid derivatives (i.e., Nonanal) and aromatic compounds (Zhang et al. 2008).

Nonanal has been reported to exhibit antimicrobial activity against gram-positive and gram-negative bacteria in the concentration of 100 to more than 800 mg/kg (Muroi et al. 1993). Nonanal is reported to have a MIC of 0.2 μg/mg against *Staphylococcus aureus* (Kubo et al. 1995). α-Phellandrene showed weak inhibitory effects against all tested bacteria at the concentrations of 1 to >4 mg/mL (Demirci et al. 2001; Iscan et al. 2012). α-Phellandrene, β-Phellandrene, ocimene, limonene, myrcene, and α-caryophyllene have shown in vitro activity against *Bacillus* sp., *Candida albicans*, *Escherichia coli*, *Pseudomonas aeruginosa*, and *S. aureus* (Perez et al. 1999; Costa et al. 2000). Essential oils are usually mixtures of monoterpene and sesquiterpene, and their oxygenated derivatives. Their composition and proportion depended on species as well as the extraction and separation methods (Fisher and Phillips 2008). Essential oils are aromatic oily liquids, their antimicrobial properties have been empirically recognized for centuries, but scientifically confirmed only recently (Dorman and Deans 2000). α-Phellandrene and Nonanal are present in large quantities in many species such as canola, soybean, *Senecio laetus*, *Haplophyllum tuberculatum*, *Minuartia meyeri* and *Apium graveolens* (Inouye et al. 2001; Fernando et al. 2005; Al-Burtamani et al. 2005; Nurettin et al. 2006; Rodriguez-Burbano et al. 2010; Hernández et al. 2013; Pandey et al. 2014). These essential oils had a broad-spectrum antimicrobial activity against various bacterials and pathogenic fungi including *Candida albicans, Alternaria alternate, Bipolaris* sp., *Curvularia lunata, Fusarium oxysporium* (Inouye et al. 2001; Al-Burtamani et al. 2005; Nurettin et al. 2006; Hernández et al. 2013; Sharma et al. 2014).

The lipophilicity of essential oils enable them to preferentially partition from an aqueous phase into membrane structures of the fungi, resulting in membrane expansion, increased membrane fluidity and permeability, disturbance of membrane-embedded proteins, inhibition of respiration, alteration of ion transport processes in fungi and induced leakage of ions and other cellular contents (Burt 2004; Fadli et al. 2012; Khan et al. 2010; Oonmetta-aree et al. 2006).

Biological activities of α-Phellandrene and Nonanal were reported only in a few publications. Further investigations are necessary to determine the anti-microbial activity of these compounds, especially for individual application, to establish the possible mechanism of action of the most active compound to combat resistant pathogenic fungi. This study aims to analyze α- Phellandrene and Nonanal on the mycelial growth of *Penicillium cyclopium*. The effects of different concentrations of α-Phellandrene and Nonanal on surface morphology, cell membrane permeability, and release of cellular material were investigated to elucidate their anti-fungal mechanisms.

## Methods

### Chemicals

α-Phellandrene (>99%) and Nonanal (>96%) were obtained from Dieckmann company (Shenzhen, China). Cholesterol (95%) and phosphovanillin (98%) were purchased from TCI Shanghai (Shanghai, China). All the chemicals were analytical grade.

### Pathogens

*Penicillium cyclopium* was provided by the Department of Biotechnology and Food Engineering, Xiangtan, China, and the fungal was isolated from infected tomato (*S. lycopersicum*) fruit. The fungal spores concentrations were adjusted to 5 × 10^5^ spores/mL using a haemocytometer before each test. 100 μL fungal suspensions (10^5^ spores/mL) were added into the triangle bottle with 40 mL potato dextrose broth (PDB) and incubated in a rotatory shaker at 28 ± 2 °C and 120 rpm for 4 days.

### Measurement of mycelial growth

Effects of α-Phellandrene and Nonanal on the mycelial growth of *P. cyclopium* were evaluated in vitro by agar dilution method (Yahyazadeh et al. 2008). PDA (20 mL) was poured to sterilized Petri dishes (90 mm diameter) and measured amounts of α-Phellandrene and Nonanal were added to PDA mediums (plus with 0.05% Tween-80) to give the following concentration of 0, 0.25, 0.50, 0.75, 1.00, 1.25, 1.50, 1.75 and 2.00 mL/L for α-Phellandrene; of 0, 50, 100, 150, 200, 250, 300, 350 and 400 μL/L for Nonanal. 6 mm diameter discs of *P. cyclopium* inocula were cut from the center of an actively growing *P. cyclopium* culture on fresh PDA plates without antibiotics at 28 ± 2 °C for 4 days with a paper punch, and then was placed at the center of each new Petri plate. The culture plates were then incubated at 28 ± 2 °C for 48 h. As controls, PDA dishes were supplemented with the same amount of filtration sterilization alcohol (99.5%) instead of α-Phellandrene and Nonanal. Each treatment was performed in triplicate. The lowest concentration that completely inhibited the growth of the fungus after 24 h of incubation was considered as the minimum inhibitory concentration (MIC). The minimum fungicidal concentration (MFC) was regarded as the lowest concentration which no growth of the pathogen was observed after a 72 h incubation period at 28 ± 2 °C in a fresh PDA plate, indicating more than 99.5% killing of the original inoculum (Talibi et al. 2012).

Different amounts of volatile substances were added to 50 ml PDB liquid medium for the final concentration of α-Phellandrene 0.00, 225.00, 450.00, 900.00, 1350.00, 1575.00 and 1800.00 μL/L, and of Nonanal 0.00, 50.00, 100.00, 200.00, 300.00, 350.00 and 400.00 μL/L. One hundred microlitres *P. cyclopium* conidial spore suspensions (10^5^ cfu/mL) were poured into each triangle bottle, which was incubated on 28 ± 2 °C and a rotatory shaker at 120 rpm for 4 days. Fungal growth was estimated gravimetrically by weighting the biomasses after drying at 80 °C to a constant weight. The percentage of mycelial growth inhibition (PGI) was calculated according to the following formula:

$$\text{PGI}(\%)=[(\text{d}_\text{c}-\text{d}_\text{t})/\text{d}_\text{c}] \times100$$

where d_c_ (g) is net dry weight of control fungi and d_t_ (g) is net dry weight of treated fungi (Helal et al. 2006).

### Release of cytoplasmic material absorbing at 260 nm

The release of cytoplasmic material absorbing at 260 nm was measured following the method of Yahyazadeh et al. (2008) and Paul et al. (2011), with some modifications. Viable cells of *P. cyclopium* in their exponential logarithmic phase were collected by vacuum filtration, then washed three times with phosphate buffered saline (pH 7.0), and re-suspended with 20 mL of the above buffer solution. The suspensions were then incubated at 28 ± 2 °C in the presence of α-Phellandrene and Nonanal at three different concentrations (0, MIC and MFC) for 0, 30, 60 and 120 min. After incubation, cells were centrifuged at 12,000*g* for 2 min, and the absorbance (260 nm) of the above supernatant (1 mL) was determined with the UV-2450 UV/Vis Spectrophotometer (Shimadzu Corporation).

### Measurement of extracellular conductivity and extracellular pH

The measurement of extracellular conductivity of *P. cyclopium* cells was conducted using a DDS-W conductivity meter (Instructions for Shanghai Precision Scientific Instrument Co., Ltd., Shanghai, China) according to the method described previously (Shao et al. 2013). The extracellular pH of *P. cyclopium* cells was determined using a Delta-320 pH-meter (Instructions for the Mettler-Toledo Co., Ltd., Shanghai, China). Initially, 100 μL fungal suspensions (10^5^ spores/mL) were added to 50 mL PDB and incubated at 28 ± 2 °C in a thermostatic cultivation shaker for 4 days. The mixtures were collected by vacuum filtration, washed for 2–3 times with sterilized double distilled water, and re-suspended in 20 mL sterilized double distilled water. After the incubation of α-Phellandrene and Nonanal at MIC or MFC for 0, 30, 60 and 120 min, the extracellular conductivity and extracellular pH of *P. cyclopium* were determined. Control flasks without α-Phellandrene or Nonanal were also tested using an equal amount of alcohol instead.

### Determination of total lipid content

Total lipid content of *P. cyclopium* cells with α-Phellandrene and Nonanal at three concentrations (0, MIC, MFC) was determined using the phosphovanillin method (Helal et al. 2007). The 3-day-old mycelia from 50 ml PDB was collected by vacuum filtration and dried with a vacuum freeze drier for 6 h. About 0.05 g of dry mycelia were homogenized with liquid nitrogen and extracted with 4.0 mL of methanol–chloroform–water mixture (2:1:0.8, v/v/v) in a clean dry test tube with vigorous shaking for 30 min. The tubes were centrifuged at 4000 rpm for 10 min. The lower phase containing lipids was thoroughly mixed with 0.2 mL saline solution and centrifuged at 4000×*g* for 10 min. Then, an aliquot of 0.2 mL chloroform and lipid mixture was transferred to a novel tube and 0.2 mL concentrated sulfuric acid (H_2_SO_4_) was added, heated for 10 min in a boiling water bath. Three milliliter phosphovanillin was added, the tube was shaken vigorously, and incubated at room temperature for 10 min. The absorbance at 520 nm was utilized to calculate total lipid contents from the standard calibration curve. Cholesterol served as a standard.

### Statistical analysis

All of the experiments were repeated three times. The results were expressed as the mean value ± standard deviation and were compared using an analysis of variance (one-way ANOVA) for multiple comparisons. All dates were processed by SPSS statistical software package release 16.0 (SPSS Inc., Chicago, IL, USA).

## Results

### Antimicrobial assay of α-Phellandrene and Nonanal

The mycelial growth of *P. cyclopium* was affected by α-Phellandrene or Nonanal in a dose-dependent manner (*P* < 0.05) (Table 1). High concentrations of α-Phellandrene (≥1.35 mL/L) or Nonanal (≥0.35 mL/L) completely inhibited the mycelial growth. The antifungal activity of Nonanal against *P. cyclopium* was more efficient than α-Phellandrene (*P* < 0.05). Low concentrations of α-Phellandrene (0.45 mL/L) or Nonanal (0.10 mL/L) only showed half of the antifungal activity against *P. cyclopium*, with PGI values of 56.90 and 57.50%, respectively. The MIC and MFC values of α-Phellandrene for *P. cyclopium* were 1.7 and 1.8 mL/L, respectively. The MIC and MFC values of Nonanal for *P. cyclopium* were 0.3 and 0.4 mL/L, respectively.

**Table 1:** Effect of α-Phellandrene and Nonanal on the mycelial growth of *Penicillium cyclopium*

α-Phellandrene concentration (μL/L)	Growth inhibition (%)^f^	Nonanal concentration (μL/L)	Growth inhibition (%)^f^
00	0.00 ± 0.00^a^	0.00	0.00 ± 0.00^a^
225.00	45.88 ± 0.56^b^	50.00	15.47 ± 0.49^b^
450.00	56.90 ± 0.64^c^	100.00	57.50 ± 1.10^c^
900.00	99.37 ± 0.01^d^	200.00	65.59 ± 0.16^d^
1350.00	100.00 ± 0.00^e^	300.00	99.61 ± 0.04^e^
1575.00	100.00 ± 0.00^e^	350.00	100.00 ± 0.00^e^
1800.00	100.00 ± 0.00^e^	400.00	100.00 ± 0.00^e^

^a–e^Significant differences at *P* < 0.05 level

^f^Values arepresented as mean ± SD

### Release of cell constituents

**Fig. 1:**
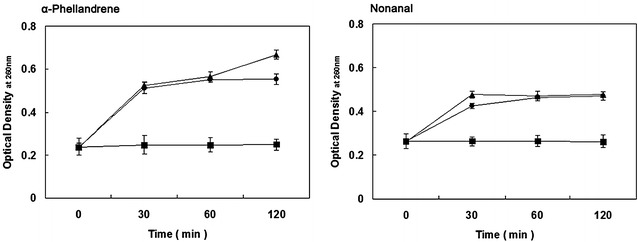
Leakage of cytoplasmic material (OD at 260 nm) from *Penicillium cyclopium* with α-Phellandrene and Nonanal treatment. (*Closed square*) control; (*closed circle*) at minimum inhibitory concentration (MIC); (*closed triangle*) at minimum fungicidal concentration (MFC). Data presented are the means of pooled data. *Error bars* indicate the SDs of the means (n = 3)

The release of cell constituents when *P. cyclopium* was treated with MIC and MFC of α-Phellandrene and Nonanal for 0, 30, 60 and 120 min are shown in Fig. 1. The optical density values (OD_260_) produced by α-Phellandrene were higher than those by Nonanal at the same treatment condition. When the *P. cyclopium* cells were treated with MIC or MFC of α-Phellandrene or Nonanal, the release of cell constituents increased. Suspensions of *P. cyclopium* with α-Phellandrene at MIC (1.7 mL/L, v/v) at 120 min reached an optical density value of 0.554, which is higher (*P* < 0.05) than that of the control (0.249), but lower than that of 0.668 at MFC (1.8 mL/L, v/v). Nevertheless, the OD_260_ values of *P. cyclopium* suspensions with α-Phellandrene treatment were almost identical before 60 min of exposure, and then increased after 60 min incubation. On the other hand, the OD_260_ values of *P. cyclopium* suspensions with Nonanal at MIC (0.3 mL/L, v/v) and MFC (0.4 mL/L, v/v) were higher than those of the control at the same exposure time. After 30 min of exposure, the OD_260_ values at MIC or MFC were almost the same.

### Scanning electron microscopy (SEM)

**Fig. 2:**
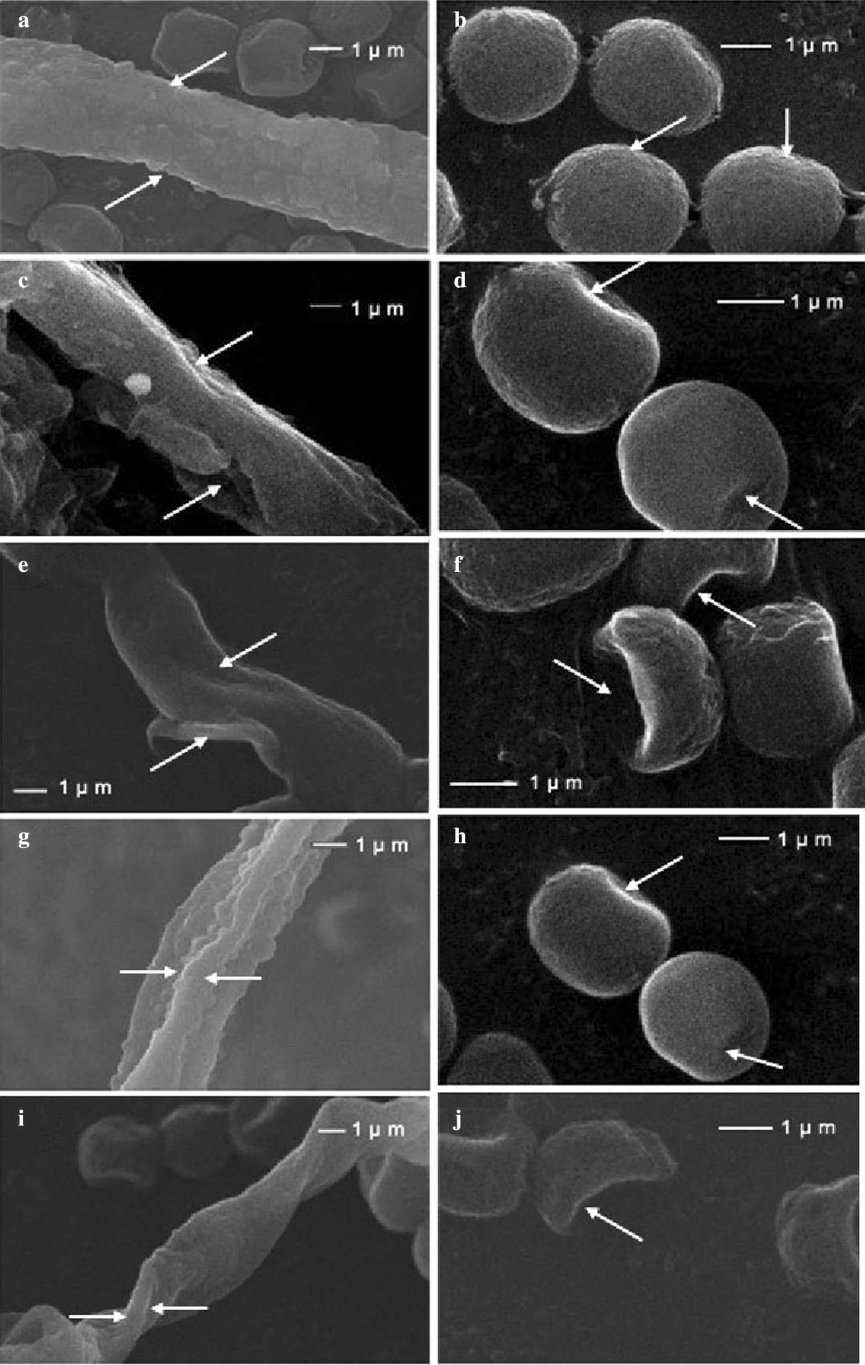
Effect of Nonanal and α-Phellandrene on mycelium and conidiophore morphological changes of *Penicillium cyclopium*. **a**, **b** *P. cyclopium* without volatile substances (control); **c**, **d** *P. cyclopium* with Nonanal at minimum inhibitory concentration (MIC); **e**, **f** *P. cyclopium* with Nonanal at minimum fungicidal concentration (MFC); **g**, **h** *P. cyclopium* with α-Phellandrene at MIC; **i**, **j** *P. cyclopium* with α-Phellandrene at MFC

The effect of Nonanal and α-Phellandrene on the morphology of *P. cyclopium* was examined using SEM (Fig. 2). The conidia of *P. cyclopium* grown on PDA plates for 4 days had normal, plump and homogenous morphology (Fig. 2b, d, f, h, j), and the hyphae of control fungus growing on PDA were normal, linearly tubular, regular, and homogeneous (Fig. 2a, c, e, g, i). The growth of *P. cyclopium* on PDA with α-Phellandrene or Nonanal at MIC or MFC treatment for 2 days demonstrated that all fungal mycelia and conidia showed considerable changes in morphology. *P. cyclopium* with Nonanal or α-Phellandrene at MIC concentrations showed slightly depressed conidia, partly distorted and shrunken mycelia (Fig. 2c, d, g, h). In contrast, the conidia of *P. cyclopium* treated with Nonanal and α-Phellandrene at MFC concentrations appeared severely collapsed and depressed possibly because of the lack of cytoplasm (Fig. 2f, j). Moreover, severely shrunken and distorted hyphae were also observed with Nonanal and α-Phellandrene at MFC concentrations (Fig. 2e, i).

### Extracellular conductivity

**Fig. 3:**
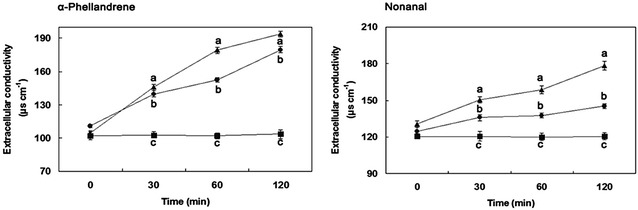
Effects of α-Phellandrene and Nonanal on the extracellular conductivity of *P. cyclopium* suspensions. (*closed square*) control; (*closed circle*) at minimum inhibitory concentration (MIC); (*closed triangle*) at minimum fungicidal concentration (MFC). Data presented are the means of pooled data. *Error bars* indicate the SDs of the means (n = 3). *Different letters* of *a*–*c* are significantly different according to Duncan’s multiple range test at *P* < 0.05

Exposure of *P. cyclopium* cells to different concentrations of α-Phellandrene and Nonanal for 0–120 min caused varying levels of extracellular conductivity (Fig. 3). In general, conductivity increased with exposure time and concentrations of α-Phellandrene and Nonanal. The concentrations for α-Phellandrene at their MIC (1.7 mL/L) and their MFC (1.8 mL/L) significantly affected the extracellular conductivity of *P. cyclopium* cells. Nevertheless, the concentrations for Nonanal at MIC (0.3 mL/L) and at MFC (0.4 mL/L) were only slightly affected with the extension of processing time. At 30 min of exposure, the extracellular conductivity of *P. cyclopium* suspensions with α-Phellandrene at MIC or MFC remained at almost the same levels, but the values were significantly higher (*P* < 0.05) than the control. By contrast, the conductivity of *P. cyclopium* suspensions with Nonanal at MFC at 30 min of exposure was 150.3 μs/cm, which was significantly higher than that at MIC (135.8 μs/cm) and that in the control (120.5 μs/cm). After 60 min of exposure, the conductivity of *P. cyclopium* suspensions treated with Nonanal remained at a stable increase. On the contrary, the conductivity of *P. cyclopium* suspensions in the presence of α-Phellandrene was markedly increased after 60 min of exposure at MFC. At 120 min, the conductivity of *P. cyclopium* suspensions at MIC reached 179.6 and 193.7 μs/cm for MFC, respectively, which were significantly higher (*P* < 0.05) than those treated with Nonanal for MIC and MFC (145.3 and 178.2 μs/cm, respectively).

### Extracellular pH

**Fig. 4:**
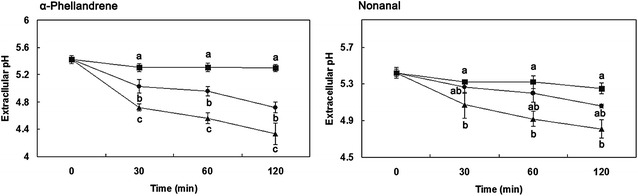
Effects of α-Phellandrene and Nonanal on the extracellular pH of *P. cyclopium* (*closed square*): control; (*closed circle*): minimum inhibitory concentration (MIC); (*closed triangle*) minimum fungicidal concentration (MFC). Data presented are the means of pooled data. *Error bars* indicate the SDs of the means (n = 3). *Different letters* of *a*–*c* are significantly different according to Duncan’s multiple range test at *P* < 0.05

The extracellular pH of *P. cyclopium* cells exposed to α-Phellandrene and Nonanal is decreased in comparison to the controls (Fig. 4). A gradual decrease in extracellular pH was observed in the control. After 30 min of exposure to α-Phellandrene at MIC and MFC, a sharp reduction in the extracellular pH of the *P. cyclopium* suspensions occurred, whereas the extracellular pH values in the *P. cyclopium* suspensions after incubation with Nonanal gradually decreased. The extracellular pH values in the *P. cyclopium* suspensions after incubation for 120 min with α-Phellandrene at MIC and MFC were 4.72 and 4.33, respectively, which were significantly lower than that of the control (5.3). No significant difference (*P* < 0.05) was found between MIC and MFC after 30 min of exposure, the extracellular pH values in *P. cyclopium* suspensions after incubation with Nonanal at MIC and MFC were 5.06 and 4.81, respectively, and the latter was significantly lower than that of the control (5.25) (*P* < 0.05).

### Potassium ion efflux

**Fig. 5:**
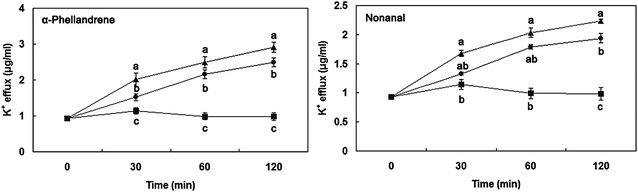
Effect of α-Phellandrene and Nonanal on the leakage of potassium ions from *P. cyclopium*. (*closed square*) control, (*closed circle*) minimum inhibitory concentration (MIC), (*closed triangle*) minimum fungicidal concentration (MFC). Data presented are the means of pooled data. *Error bars* indicate the SDs of the means (n = 3). *Different letters* of *a*–*c* are significantly different according to Duncan’s multiple range test at *P* < 0.05

Potassium ions (K^+^) leaked from *P. cyclopium* cells incubated with α-Phellandrene and Nonanal (Fig. 5). MFC and MIC of α-Phellandrene and Nonanal significantly induced the release of K^+^, and the K^+^ concentration after 30 min was 1.520 and 1.330 μg/mL, respectively. When the incubation time increased to 120 min, the release of K^+^ concentration continuously increased. By contrast, incubation with MFC of α-Phellandrene and Nonanal resulted in more K^+^ release than from the *P. cyclopium* cells with MIC of those. After 120 min of incubation, K^+^ released by MFC of α-Phellandrene and Nonanal reached 2.910 and 2.235 μg/mL, respectively.

### Total lipid content

**Fig. 6:**
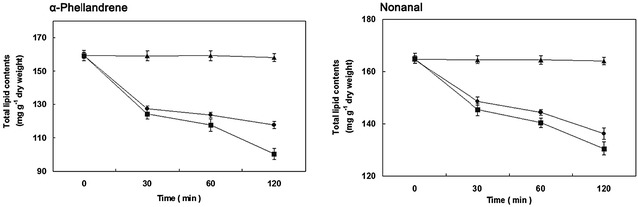
Total lipid content of *P. cyclopium* cells in the presence of α-Phellandrene and Nonanal. (*closed square*) control, (*closed circle*) at minimum inhibitory concentration (MIC), (*closed triangle*) at minimum fungicidal concentration (MFC). Data presented are the means of pooled data. *Error bars* indicate the SDs of the means (n = 3)

α-Phellandrene and Nonanal affected the total lipid contents of *P. cyclopium* cells (Fig. 6). Briefly, α-Phellandrene and Nonanal significantly decreased the total lipid content, especially for α-Phellandrene (*P* < 0.05). The total lipid contents of the *P. cyclopium* cells were 100.3 ± 2.4 and 117.4 ± 2.1 mg/g dry weight for 120 min after incubation with α-Phellandrene at MFC and MIC, respectively. These values are significantly lower (*P* < 0.05) than those of the control (158.1 ± 2.3 mg/g dry weight). By contrast, Nonanal at MFC and MIC remarkably reduced the total lipid content of *P. cyclopium* cells. Moreover, the effect of Nonanal at MFC and MIC was lower than that of α-Phellandrene in *P. cyclopium* cells. The total lipid contents of the *P. cyclopium* cells were 130.5 ± 2.4 and 136.2 ± 3.2 mg/g dry weight, respectively, after incubation with Nonanal at MFC and MIC for 120 min.

## Discussion

α-Phellandrene and Nonanal exhibited strong antifungal activity against *P. cyclopium*. The inhibitory effect was positively correlated with the concentration of α-Phellandrene and Nonanal. These results were consistent with those of previous studies describing the antifungal activity of these volatile compounds (Fernando et al. 2005; Rodriguez-Burbano et al. 2010; Pandey et al. 2014; Sharma et al. 2014). At a relatively low concentration (0.1 mL/L), Nonanal reduced the mycelial growth of *P. cyclopium* by half, making it a promising antifungal substance. In addition, the inhibitory effect of Nonanal on *P. cyclopium* was more efficient than that of α-Phellandrene on *P. cyclopium*. The phenomenons were not observed in α-Phellandrene and Nonanal, indicating that the aldehyde compounds are more effective than alcohols and olefine in controlling postharvest pathogens (Droby et al. 2008). Among aldehyde constituents, cinnamaldehyde showed the highest activity, followed by citral, and then perillaldehyde, octanal and Nonanal (Inouye et al. 2001).

The potential mechanisms underlying the anti-microbial activity of aldehydes and terpenes are not fully understood, but a number of possible mechanisms have been proposed. Gram-positive bacteria are known to be more susceptible to essential oils than Gram-negative bacteria (Farag et al. 1989; Smith-Palmer et al. 1998). The weak antibacterial activity against Gram-negative bacteria was ascribed to the presence of an outer membrane (Tassou and Nychas 1995; Mann et al. 2000), which possessed hydrophilic polysaccharide chains as a barrier to hydrophobic essential oils. In the current experiment, in vitro antifungal activity enabled us to hypothesize that the potential antifungal activity of α-Phellandrene and Nonanal against *P. cyclopium* could be closely correlated with the physiology of the hyphae. SEM analysis showed that the volatile compounds could alter the morphology of *P. cyclopium* hyphae, disrupting the membrane integrity (Yahyazadeh et al. 2008; Tyagi and Malik, 2011).

These changes generally occur because of an increase in the permeability of cells, and such changes commonly result in the leakage of small molecular substances, ions, and formation of lesions (Bajpai et al. 2013). The leakage of cytoplasmic membrane was analyzed by determining the release of cell materials including nucleic acid, metabolites and ions which was absorbed at 260 nm in the suspensions (Oonmetta-aree et al. 2006). After the addition of the α-Phellandrene and Nonanal visibly increased with increasing volatile compound concentration. The maximum release of cell constituents was observed in *P. cyclopium* treated with α-Phellandrene at MFC. The methyl ester is able to penetrate to the hydrophobic regions of the membranes and the carboxyl groups pass through the cell membrane, perturbing in the lowering of internal pH and denaturing of proteins inside the cell (Marquis et al. 2003). From the results of the present study combined with the previous studies, we can conclude that the two volatile compounds apparently induced the leakage of intracellular protons. These findings suggest that irreversible damage to the cytoplasmic membranes of *P. cyclopium* occurred, and the ions inside the cells leaked, ultimately leading to apoptosis of the fungus in the presence of volatile compounds.

In addition to cell wall and plasma membrane alteration and disruption, exposure of the hyphae of *P. cyclopium* to α-Phellandrene or Nonanal resulted in K^+^ leakage. Our results are in agreement with the reported by Helal et al. (2006). The phenomenon could be explained that the release of ions was only based on their size and/or due to formation of holes or lesions of lipid bilayer of the plasma membrane (Prashar et al. 2003). The fatty acid composition of microbial cell membranes affects their ability to survive in various environments (Ghfir et al. 1994). The decrease in lipid content suggested that membrane stability decreased while the permeability of water-soluble materials increased (Helal et al. 2007). Fumigation of *P. cyclopium* with *C. citratus* essential oil, induced alterations in both the lipid content and the fatty acids methyl esters composition of the cells (Helal et al. 2006). In the present study, the addition of α-Phellandrene and Nonanal significantly decreased the lipid content of *P. cyclopium*. The results have shown that the two volatile compounds had the ability to penetrate lipid structures of the cells and disrupt the cell membrane integrity.

In conclusion, this study showed that α-Phellandrene and Nonanal could significantly inhibit the mycelial growth of *P. cyclopium* cells, disrupt their cell membrane integrity and result in the leakage of cell components. Our present study suggested that α-Phellandrene and Nonanal might be used as fungicides to fight against postharvest fungal diseases.

## Conclusions

α-Phellandrene and Nonanal significantly inhibit the mycelia growth of *P. cyclopium*. These changes disrupt the integrity of the fungal cell membrane, leading to the leakage of cell constituent and potassium ions, and triggering an increase of the total lipid content, extracellular pH and membrane permeability. α-Phellandrene and Nonanal might be used as biological fungicides for the control of *P. cyclopium* in postharvest tomato fruits in the future.
